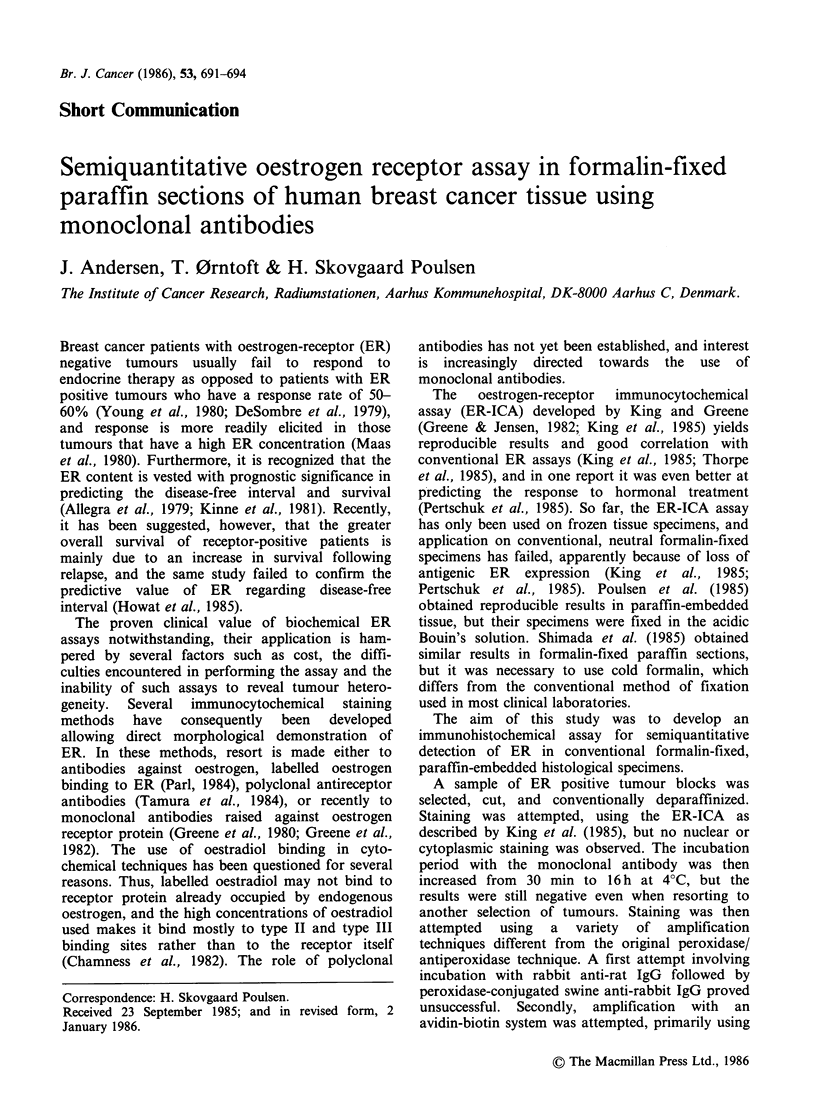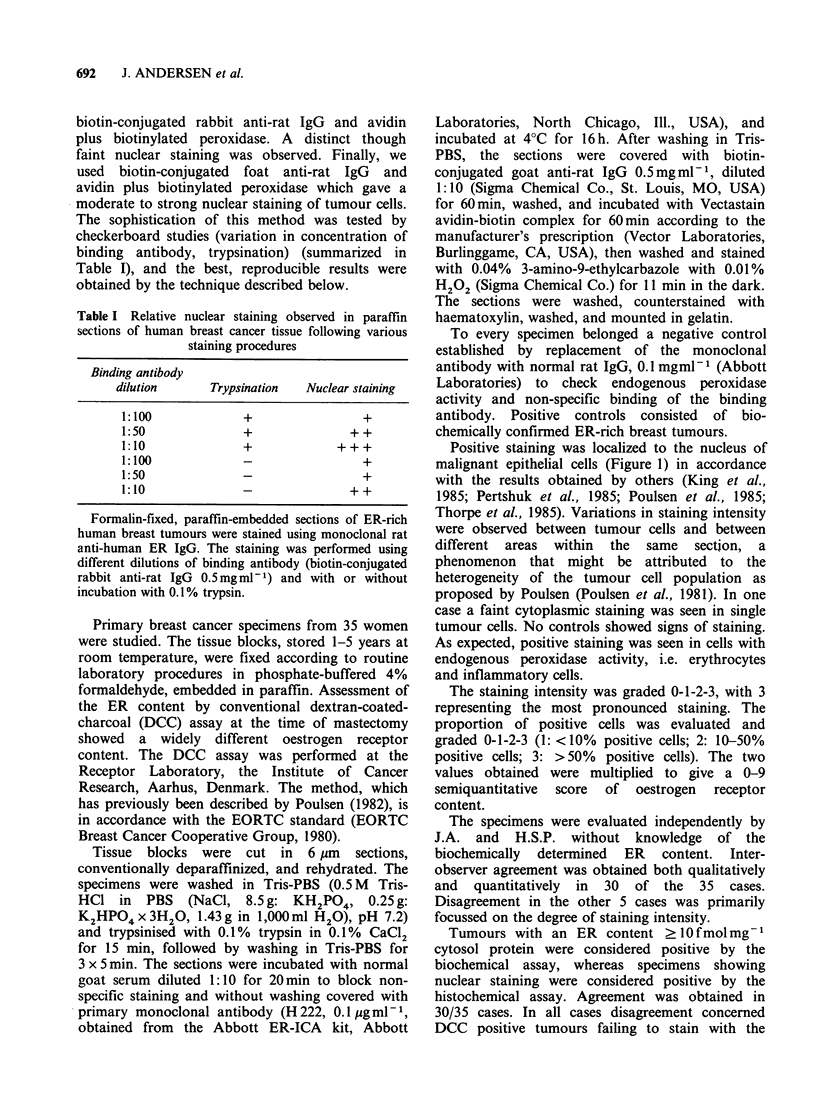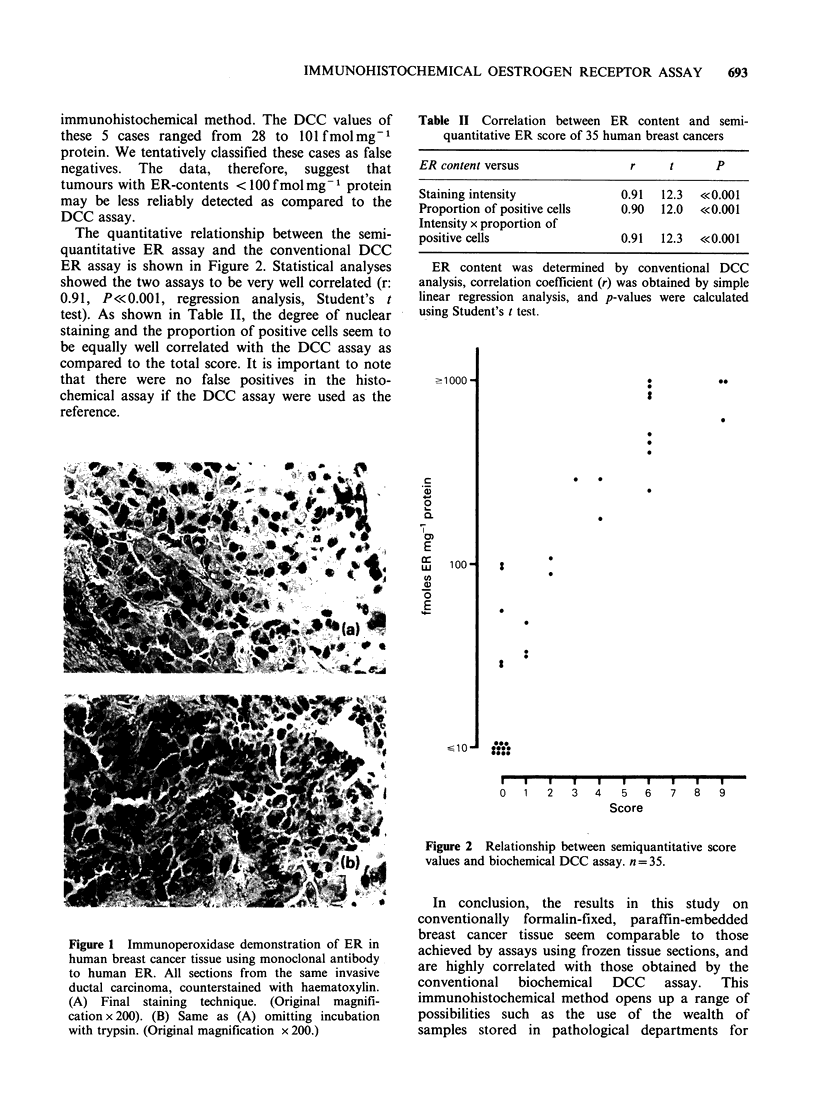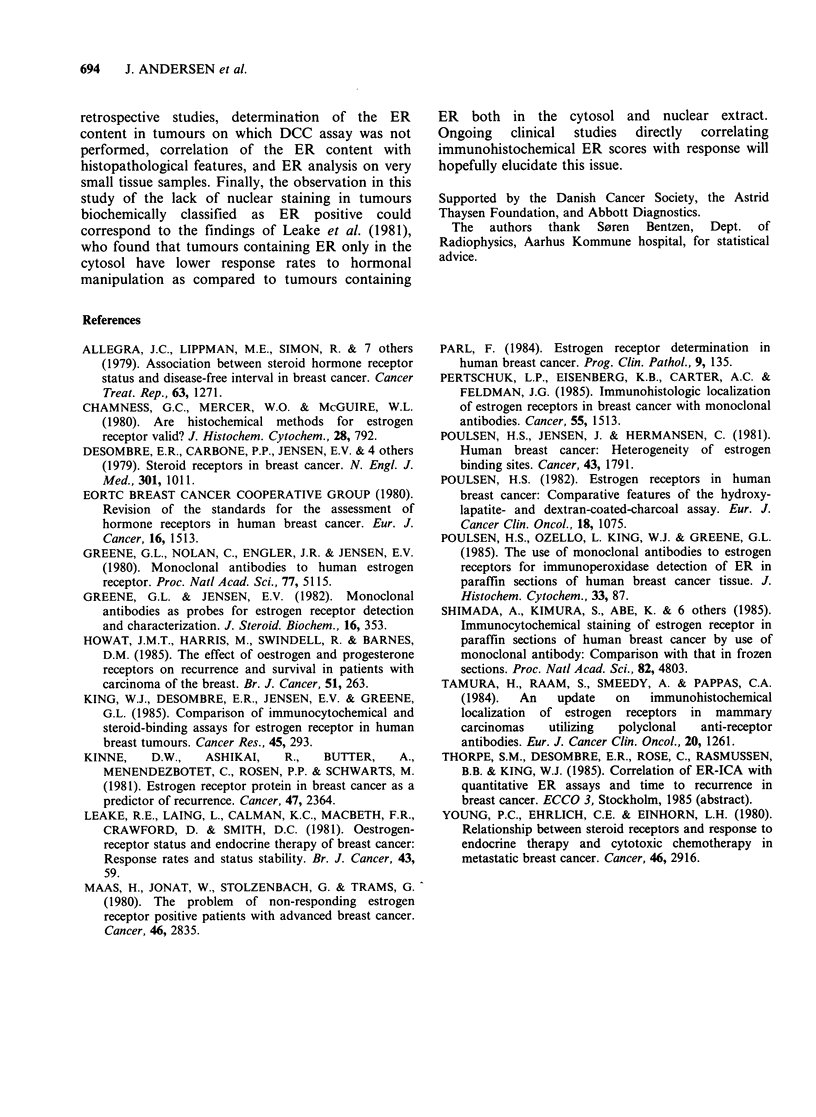# Semiquantitative oestrogen receptor assay in formalin-fixed paraffin sections of human breast cancer tissue using monoclonal antibodies.

**DOI:** 10.1038/bjc.1986.114

**Published:** 1986-05

**Authors:** J. Andersen, T. Orntoft, H. S. Poulsen

## Abstract

**Images:**


*Br. J. Cancer* (1986), **53**, 691–694

### Short Communication

### Semiquantitative oestrogen receptor assay in formalin-fixed paraffin sections of human breast cancer tissue using monoclonal antibodies

J. Andersen, T. Ørntoft & H. Skovgaard Poulsen

*The Institute of Cancer Research, Radiumstationen, Aarhus Kommunehospital, DK-8000 Aarhus C, Denmark.*

Breast cancer patients with oestrogen-receptor (ER) negative tumours usually fail to respond to endocrine therapy as opposed to patients with ER positive tumours who have a response rate of 50–60% (Young *et al.*, 1980; DeSombre *et al.*, 1979), and response is more readily elicited in those tumours that have a high ER concentration (Maas *et al.*, 1980). Furthermore, it is recognized that the ER content is vested with prognostic significance in predicting the disease-free interval and survival (Allegra *et al.*, 1979; Kinne *et al.*, 1981). Recently, it has been suggested, however, that the greater overall survival of receptor-positive patients is mainly due to an increase in survival following relapse, and the same study failed to confirm the predictive value of ER regarding disease-free interval (Howat *et al.*, 1985).

The proven clinical value of biochemical ER assays notwithstanding, their application is hampered by several factors such as cost, the difficulties encountered in performing the assay and the inability of such assays to reveal tumour heterogeneity. Several immunocytochemical staining methods have consequently been developed allowing direct morphological demonstration of ER. In these methods, resort is made either to antibodies against oestrogen, labelled oestrogen binding to ER (Parl, 1984), polyclonal antireceptor antibodies (Tamura *et al.*, 1984), or recently to monoclonal antibodies raised against oestrogen receptor protein (Greene *et al.*, 1980; Greene *et al.*, 1982). The use of oestradiol binding in cytochemical techniques has been questioned for several reasons. Thus, labelled oestradiol may not bind to receptor protein already occupied by endogenous oestrogen, and the high concentrations of oestradiol used makes it bind mostly to type II and type III binding sites rather than to the receptor itself (Chamness *et al.*, 1982). The role of polyclonal antibodies has not yet been established, and interest is increasingly directed towards the use of monoclonal antibodies.

The oestrogen-receptor immunocytochemical assay (ER-ICA) developed by King and Greene (Greene & Jensen, 1982; King *et al.*, 1985) yields reproducible results and good correlation with conventional ER assays (King *et al.*, 1985; Thorpe *et al.*, 1985), and in one report it was even better at predicting the response to hormonal treatment (Pertschuk *et al.*, 1985). So far, the ER-ICA assay has only been used on frozen tissue specimens, and application on conventional, neutral formalin-fixed specimens has failed, apparently because of loss of antigenic ER expression (King *et al.*, 1985; Pertschuk *et al.*, 1985). Poulsen *et al.* (1985) obtained reproducible results in paraffin-embedded tissue, but their specimens were fixed in the acidic Bouin's solution. Shimada *et al.* (1985) obtained similar results in formalin-fixed paraffin sections, but it was necessary to use cold formalin, which differs from the conventional method of fixation used in most clinical laboratories.

The aim of this study was to develop an immunohistochemical assay for semiquantitative detection of ER in conventional formalin-fixed, paraffin-embedded histological specimens.

A sample of ER positive tumour blocks was selected, cut, and conventionally deparaffinized. Staining was attempted, using the ER-ICA as described by King *et al.* (1985), but no nuclear or cytoplasmic staining was observed. The incubation period with the monoclonal antibody was then increased from 30 min to 16 h at 4°C, but the results were still negative even when resorting to another selection of tumours. Staining was then attempted using a variety of amplification techniques different from the original peroxidase/antiperoxidase technique. A first attempt involving incubation with rabbit anti-rat IgG followed by peroxidase-conjugated swine anti-rabbit IgG proved unsuccessful. Secondly, amplification with an avidin-biotin system was attempted, primarily using

Correspondence: H. Skovgaard Poulsen.
Received 23 September 1985; and in revised form, 2 January 1986.

© The Macmillan Press Ltd., 1986

biotin-conjugated rabbit anti-rat IgG and avidin plus biotinylated peroxidase. A distinct though faint nuclear staining was observed. Finally, we used biotin-conjugated foat anti-rat IgG and avidin plus biotinylated peroxidase which gave a moderate to strong nuclear staining of tumour cells. The sophistication of this method was tested by checkerboard studies (variation in concentration of binding antibody, trypsination) (summarized in Table I), and the best, reproducible results were obtained by the technique described below.

**Table I** Relative nuclear staining observed in paraffin sections of human breast cancer tissue following various staining procedures

| *Binding antibody dilution* | *Trypsination* | *Nuclear staining* |
|---|---|---|
| 1:100 | + | + |
| 1:50 | + | ++ |
| 1:10 | + | +++ |
| 1:100 | − | + |
| 1:50 | − | + |
| 1:10 | − | ++ |

Formalin-fixed, paraffin-embedded sections of ER-rich human breast tumours were stained using monoclonal rat anti-human ER IgG. The staining was performed using different dilutions of binding antibody (biotin-conjugated rabbit anti-rat IgG $0.5\,mg\,ml^{-1}$) and with or without incubation with 0.1% trypsin.

Primary breast cancer specimens from 35 women were studied. The tissue blocks, stored 1–5 years at room temperature, were fixed according to routine laboratory procedures in phosphate-buffered 4% formaldehyde, embedded in paraffin. Assessment of the ER content by conventional dextran-coated-charcoal (DCC) assay at the time of mastectomy showed a widely different oestrogen receptor content. The DCC assay was performed at the Receptor Laboratory, the Institute of Cancer Research, Aarhus, Denmark. The method, which has previously been described by Poulsen (1982), is in accordance with the EORTC standard (EORTC Breast Cancer Cooperative Group, 1980).

Tissue blocks were cut in 6 $\mu m$ sections, conventionally deparaffinized, and rehydrated. The specimens were washed in Tris-PBS (0.5 M Tris-HCl in PBS (NaCl, 8.5 g: $KH_2PO_4$, 0.25 g: $K_2HPO_4 \times 3H_2O$, 1.43 g in 1,000 ml $H_2O$), pH 7.2) and trypsinised with 0.1% trypsin in 0.1% $CaCl_2$ for 15 min, followed by washing in Tris-PBS for 3 x 5 min. The sections were incubated with normal goat serum diluted 1:10 for 20 min to block non-specific staining and without washing covered with primary monoclonal antibody (H222, $0.1\,\mu g\,ml^{-1}$, obtained from the Abbott ER-ICA kit, Abbott Laboratories, North Chicago, Ill., USA), and incubated at 4°C for 16 h. After washing in Tris-PBS, the sections were covered with biotin-conjugated goat anti-rat IgG $0.5\,mg\,ml^{-1}$, diluted 1:10 (Sigma Chemical Co., St. Louis, MO, USA) for 60 min, washed, and incubated with Vectastain avidin-biotin complex for 60 min according to the manufacturer's prescription (Vector Laboratories, Burlinggame, CA, USA), then washed and stained with 0.04% 3-amino-9-ethylcarbazole with 0.01% $H_2O_2$ (Sigma Chemical Co.) for 11 min in the dark. The sections were washed, counterstained with haematoxylin, washed, and mounted in gelatin.

To every specimen belonged a negative control established by replacement of the monoclonal antibody with normal rat IgG, $0.1\,mg\,ml^{-1}$ (Abbott Laboratories) to check endogenous peroxidase activity and non-specific binding of the binding antibody. Positive controls consisted of biochemically confirmed ER-rich breast tumours.

Positive staining was localized to the nucleus of malignant epithelial cells (Figure 1) in accordance with the results obtained by others (King *et al.*, 1985; Pertshuk *et al.*, 1985; Poulsen *et al.*, 1985; Thorpe *et al.*, 1985). Variations in staining intensity were observed between tumour cells and between different areas within the same section, a phenomenon that might be attributed to the heterogeneity of the tumour cell population as proposed by Poulsen (Poulsen *et al.*, 1981). In one case a faint cytoplasmic staining was seen in single tumour cells. No controls showed signs of staining. As expected, positive staining was seen in cells with endogenous peroxidase activity, i.e. erythrocytes and inflammatory cells.

The staining intensity was graded 0-1-2-3, with 3 representing the most pronounced staining. The proportion of positive cells was evaluated and graded 0-1-2-3 (1: <10% positive cells; 2: 10–50% positive cells; 3: >50% positive cells). The two values obtained were multiplied to give a 0–9 semiquantitative score of oestrogen receptor content.

The specimens were evaluated independently by J.A. and H.S.P. without knowledge of the biochemically determined ER content. Inter-observer agreement was obtained both qualitatively and quantitatively in 30 of the 35 cases. Disagreement in the other 5 cases was primarily focussed on the degree of staining intensity.

Tumours with an ER content $\geq 10\,fmol\,mg^{-1}$ cytosol protein were considered positive by the biochemical assay, whereas specimens showing nuclear staining were considered positive by the histochemical assay. Agreement was obtained in 30/35 cases. In all cases disagreement concerned DCC positive tumours failing to stain with the immunohistochemical method. The DCC values of these 5 cases ranged from 28 to 101 $fmol\,mg^{-1}$ protein. We tentatively classified these cases as false negatives. The data, therefore, suggest that tumours with ER-contents $<100\,fmol\,mg^{-1}$ protein may be less reliably detected as compared to the DCC assay.

The quantitative relationship between the semi-quantitative ER assay and the conventional DCC ER assay is shown in Figure 2. Statistical analyses showed the two assays to be very well correlated (r: 0.91, $P \ll 0.001$, regression analysis, Student's $t$ test). As shown in Table II, the degree of nuclear staining and the proportion of positive cells seem to be equally well correlated with the DCC assay as compared to the total score. It is important to note that there were no false positives in the histochemical assay if the DCC assay were used as the reference.

**Figure 1** Immunoperoxidase demonstration of ER in human breast cancer tissue using monoclonal antibody to human ER. All sections from the same invasive ductal carcinoma, counterstained with haematoxylin. (A) Final staining technique. (Original magnification × 200). (B) Same as (A) omitting incubation with trypsin. (Original magnification × 200.)

**Table II** Correlation between ER content and semi-quantitative ER score of 35 human breast cancers

| *ER content* versus | $r$ | $t$ | $P$ |
|---|---|---|---|
| Staining intensity | 0.91 | 12.3 | ≪0.001 |
| Proportion of positive cells | 0.90 | 12.0 | ≪0.001 |
| Intensity × proportion of positive cells | 0.91 | 12.3 | ≪0.001 |

ER content was determined by conventional DCC analysis, correlation coefficient ($r$) was obtained by simple linear regression analysis, and $p$-values were calculated using Student's $t$ test.

**Figure 2** Relationship between semiquantitative score values and biochemical DCC assay. $n=35$.

In conclusion, the results in this study on conventionally formalin-fixed, paraffin-embedded breast cancer tissue seem comparable to those achieved by assays using frozen tissue sections, and are highly correlated with those obtained by the conventional biochemical DCC assay. This immunohistochemical method opens up a range of possibilities such as the use of the wealth of samples stored in pathological departments for retrospective studies, determination of the ER content in tumours on which DCC assay was not performed, correlation of the ER content with histopathological features, and ER analysis on very small tissue samples. Finally, the observation in this study of the lack of nuclear staining in tumours biochemically classified as ER positive could correspond to the findings of Leake *et al.* (1981), who found that tumours containing ER only in the cytosol have lower response rates to hormonal manipulation as compared to tumours containing ER both in the cytosol and nuclear extract. Ongoing clinical studies directly correlating immunohistochemical ER scores with response will hopefully elucidate this issue.

Supported by the Danish Cancer Society, the Astrid Thaysen Foundation, and Abbott Diagnostics.

The authors thank Søren Bentzen, Dept. of Radiophysics, Aarhus Kommune hospital, for statistical advice.

### References

ALLEGRA, J.C., LIPPMAN, M.E., SIMON, R. & 7 others (1979). Association between steroid hormone receptor status and disease-free interval in breast cancer. *Cancer Treat. Rep.*, **63**, 1271.

CHAMNESS, G.C., MERCER, W.O. & McGUIRE, W.L. (1980). Are histochemical methods for estrogen receptor valid? *J. Histochem. Cytochem.*, **28**, 792.

DESOMBRE, E.R., CARBONE, P.P., JENSEN, E.V. & 4 others (1979). Steroid receptors in breast cancer. *N. Engl. J. Med.*, **301**, 1011.

EORTC BREAST CANCER COOPERATIVE GROUP (1980). Revision of the standards for the assessment of hormone receptors in human breast cancer. *Eur. J. Cancer*, **16**, 1513.

GREENE, G.L., NOLAN, C., ENGLER, J.R. & JENSEN, E.V. (1980). Monoclonal antibodies to human estrogen receptor. *Proc. Natl Acad. Sci.*, **77**, 5115.

GREENE, G.L. & JENSEN, E.V. (1982). Monoclonal antibodies as probes for estrogen receptor detection and characterization. *J. Steroid. Biochem.*, **16**, 353.

HOWAT, J.M.T., HARRIS, M., SWINDELL, R. & BARNES, D.M. (1985). The effect of oestrogen and progesterone receptors on recurrence and survival in patients with carcinoma of the breast. *Br. J. Cancer*, **51**, 263.

KING, W.J., DESOMBRE, E.R., JENSEN, E.V. & GREENE, G.L. (1985). Comparison of immunocytochemical and steroid-binding assays for estrogen receptor in human breast tumours. *Cancer Res.*, **45**, 293.

KINNE, D.W., ASHIKAI, R., BUTTER, A., MENENDEZBOTET, C., ROSEN, P.P. & SCHWARTS, M. (1981). Estrogen receptor protein in breast cancer as a predictor of recurrence. *Cancer*, **47**, 2364.

LEAKE, R.E., LAING, L., CALMAN, K.C., MACBETH, F.R., CRAWFORD, D. & SMITH, D.C. (1981). Oestrogen-receptor status and endocrine therapy of breast cancer: Response rates and status stability. *Br. J. Cancer*, **43**, 59.

MAAS, H., JONAT, W., STOLZENBACH, G. & TRAMS, G. (1980). The problem of non-responding estrogen receptor positive patients with advanced breast cancer. *Cancer*, **46**, 2835.

PARL, F. (1984). Estrogen receptor determination in human breast cancer. *Prog. Clin. Pathol.*, **9**, 135.

PERTSCHUK, L.P., EISENBERG, K.B., CARTER, A.C. & FELDMAN, J.G. (1985). Immunohistologic localization of estrogen receptors in breast cancer with monoclonal antibodies. *Cancer*, **55**, 1513.

POULSEN, H.S., JENSEN, J. & HERMANSEN, C. (1981). Human breast cancer: Heterogeneity of estrogen binding sites. *Cancer*, **43**, 1791.

POULSEN, H.S. (1982). Estrogen receptors in human breast cancer: Comparative features of the hydroxylapatite- and dextran-coated-charcoal assay. *Eur. J. Cancer Clin. Oncol.*, **18**, 1075.

POULSEN, H.S., OZELLO, L. KING, W.J. & GREENE, G.L. (1985). The use of monoclonal antibodies to estrogen receptors for immunoperoxidase detection of ER in paraffin sections of human breast cancer tissue. *J. Histochem. Cytochem.*, **33**, 87.

SHIMADA, A., KIMURA, S., ABE, K. & 6 others (1985). Immunocytochemical staining of estrogen receptor in paraffin sections of human breast cancer by use of monoclonal antibody: Comparison with that in frozen sections. *Proc. Natl Acad. Sci.*, **82**, 4803.

TAMURA, H., RAAM, S., SMEEDY, A. & PAPPAS, C.A. (1984). An update on immunohistochemical localization of estrogen receptors in mammary carcinomas utilizing polyclonal anti-receptor antibodies. *Eur. J. Cancer Clin. Oncol.*, **20**, 1261.

THORPE, S.M., DESOMBRE, E.R., ROSE, C., RASMUSSEN, B.B. & KING, W.J. (1985). Correlation of ER-ICA with quantitative ER assays and time to recurrence in breast cancer. *ECCO 3*, Stockholm, 1985 (abstract).

YOUNG, P.C., EHRLICH, C.E. & EINHORN, L.H. (1980). Relationship between steroid receptors and response to endocrine therapy and cytotoxic chemotherapy in metastatic breast cancer. *Cancer*, **46**, 2916.